# Is Evolution of Mating Preferences Inevitable? Random Mating in the Multisex System of *Tetrahymena thermophila*

**DOI:** 10.1155/2012/201921

**Published:** 2012-09-27

**Authors:** Sujal S. Phadke, Lauren Cooper, Rebecca A. Zufall

**Affiliations:** ^1^Department of Biology and Biochemistry, University of Houston, Houston, TX 77204, USA; ^2^Department of Molecular Genetics and Microbiology, Duke University Medical Center, Durham, NC 27710, USA

## Abstract

Ciliate mating systems are highly diversified, providing unique opportunities to study sexual differentiation and its implications for mating dynamics. Many species of ciliates have multiple (>2) sexes. More sexes may mean more choice and an opportunity for evolution of preferential mating. We asked if the multiple sexes of the ciliate *Tetrahymena thermophila* mate preferentially among each other. We quantified pairing frequencies among four sexes of *T. thermophila* using experiments that allowed the sexes to compete as mating partners. We found that all sexes mated equally frequently among each other, that is, we found no evidence of preferential mating with respect to sex. This suggests that the “mate choice” in this ciliate is binary, between whether to form a pair or not and, in this regard, sex facilitates only self-/non-self-distinction. Thus, presence of multiple sexes does not necessarily result in the evolution of mating bias, which could decrease the maximum amount of mating that would otherwise be possible in a population. Our result of random mating verifies a key assumption in the theoretical model of sex ratio evolution in *T. thermophila*. Investigation into molecular differences between the sexes will be necessary to reveal the mechanistic basis of random mating among them.

## 1. Introduction

Mating is random when two individuals in a population are just as likely as any other two individuals to mate. Evolution of mating preferences requires that potential mates are differentially attractive. Thus, random mating is expected if there is little variance in the perceived “quality” of mates. In natural populations, mating is rarely random [1]. 

Nonrandom mating results when individuals tend to choose mates with a specific phenotype and the associated genotype(s) among compatible mates. Dynamics of nonrandom mating have been studied in sexually dimorphic species in which size, sound, and color often describe the most preferred phenotype [2]. Among the microbial eukaryotes, mate-preference has been demonstrated in the yeast *Saccharomyces cerevisiae*, in which the highest amount of pheromone produced defines the most preferred phenotype for the cells of either sex [3]. It is largely unclear how mates are chosen in other unicellular organisms. Often, unicellular species have more than two sexes, raising an obvious yet previously unanswered question: do more sexes mean more choice, thereby making evolution of mate preference among the sexes inevitable? In other words, is selective mating observed when there is an opportunity to choose between many compatible sexes? For instance, the multiple sexes of a species could form a hierarchy from the best phenotype (the most preferred sex) to the least preferred one. Alternatively, the multiple sexes could be grouped such that sexes within a group mate more frequently with each other than those between groups, resulting in pronounced mating preferences between groups. 

The ciliate *Tetrahymena thermophila* has seven, self-incompatible mating types (sexes). Pair formation between cells of any two sexes initiates mating (conjugation) and subsequent genetic exchange. Although each of the seven sexes identified in *T. thermophila* readily forms pairs with the other six sexes [4] the frequency of pair formation between sexes, that is, the degree of selective mating, has not been quantified when more than two compatible sexes are simultaneously present in a population offering a choice of mate.

In *T. thermophila*, cells of different sexes engage in physical interactions (costimulation), which last for up to 2 hours prior to pair formation. Costimulation by one compatible sex does not block pair formation with any other sex. Also, the extent of costimulation by one compatible sex does not affect the efficiency (total amount and the “speed”) of pairing with another compatible sex [5]. Although this shows that costimulation is not sex-specific, the exact molecular interactions that occur during costimulation are still a mystery. Also, molecular differences between the seven sexes are unknown. Current speculation is that a unique glycoprotein ligand-receptor pair may characterize each sex and that the interaction between a sex-specific ligand carried by one partner with its receptor displayed on the surface of the other partner may lead to mating pair formation in *T. thermophila *[6–8]. Under this model, affinity between the ligand and receptor may determine how likely a sex is chosen as a mate, that is, pairs would more often be formed with the sex whose ligand shares the strongest affinity for the receptor. This would result in nonrandom mating frequencies for the various sexes in a population. Another way in which the molecular affinities may affect the pairing frequencies is through mating kinetics, which affect the rate of pair formation [9]. A sex whose ligand and receptor share the strongest affinity may begin pairing with the other sexes early, resulting in nonrandom mating if limited time is available to find a partner; however, given enough time, the initial differences in pairing frequencies may disappear. Mating kinetics has been previously documented to contribute to nonrandom mating in yeast [10, 11].

Here, we tested the null hypothesis that the sexes of *T. thermophila* mate randomly with respect to sex of a partner. Our experimental design allowed individuals a limited amount of time to choose between two compatible sexes (or not to mate at all), and we recorded the number of times each compatible sex was chosen as a partner. Under the conditions tested, we found that this species mates randomly with respect to sex.

## 2. Materials and Methods

### 2.1. Strains

**Figure 1:**
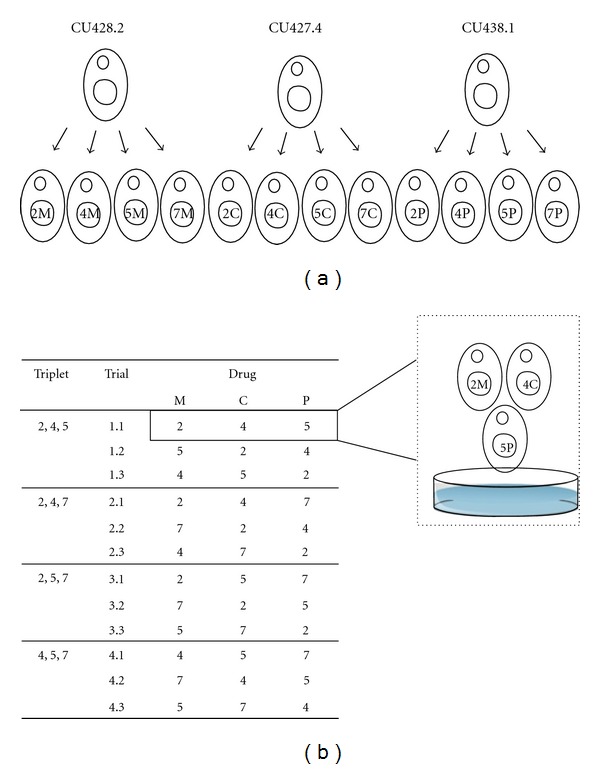
Experimental design. (a) Strain construction: three parental strains obtained from the *Tetrahymena* stock center (Table 1) were individually subjected to a genomic exclusion cross with the strain A*III [13] to construct an array of drug resistant progeny strains of sexes II, IV, V, and VII (shown here as Arabic numbers for convenience). Drugs are represented by M: 6-methylpurine; C: cyclohexamide; P: paromomycin. These 12 progeny strains (Table 1) were used to set up experimental trials. (b) Mate choice assay: for each triplet of sexes, separate trials were conducted, in which the resistant genetic backgrounds alternated between the three sexes. Thus, each trial contained a unique combination of progeny strains that were allowed to compete for mating partners.

We obtained the strains CU427.4, CU428.2, and CU438.1 (Table 1) from the *Tetrahymena* stock center (Cornell University). Each strain carries a different dominant drug resistance marker in the germline nucleus. These strains have a sensitive phenotype in all drugs because their somatic nuclei contain the drug sensitive allele (Table 1). All strains have the *mat*-2 allele at the sex determination locus [12, 14, 15]. We performed a genomic exclusion (GE) cross of each strain (following the methods in [13, 16]) with the strain A*III, which does not contribute its genome to the progeny. We obtained 4 progeny, with mating types II, IV, V, and VII, from each parental strain (Figure 1(a), Table 1). Each progeny inherits the *mat*-2 allele and expresses one of the seven sexes according to the sex determination pattern of *mat-*2 [6]. The progeny of a strain carry the same drug resistance marker as their parent strain, but in contrast to the parental strain, the progeny express the drug resistant phenotype. 

### 2.2. Culture Media

Stocks of all strains were maintained frozen under liquid nitrogen for the entire duration of the study. Frozen stocks were thawed, and cells were grown to log phase for 48 hours prior, to use in the experiments. We used 2% w/v Proteose Peptone (PP) to grow cells asexually. 1% PP was used for isolation of mating pairs. This medium, unlike 2% PP, buffers the pairs against osmotic shock, allowing completion of mating and subsequent asexual growth [16]. To induce mating, all strains were starved in 2% bacterized peptone (BP). To make 2% BP, an overnight culture of *Klebsiella pneumoniae* grown in 2% PP was diluted 1 : 50 with sterile water. In this medium, ciliates grow asexually by feeding on the bacteria and starve upon exhausting the bacteria in about 48 hours [16]. We used 2% BP, instead of conventional starvation media (e.g., 10 mM Tris), to mimic starvation in the natural environment. Also, 2%BP is the least likely to modify the molecular interactions and influence mating propensities between different sexes. The starved cells were washed and all matings were performed in autoclaved distilled water.

### 2.3. Identification of Sex

Mating type (MT) tests are used to identify mating type (sex) of a new progeny cell produced as a result of a cross [16]. Self-incompatibility, which is the inability of cells to form pairs with other cells of the same sex, is a key property used in MT tests. When mixed separately with a culture of each of the seven sexes, a clonal culture forms pairs with all but one of the seven mating type tester strains. Absence of pairing is interpreted as evidence that the progeny culture has the same sex as the tester strain. We used this protocol to determine the sex of each progeny strain generated by genomic exclusion (Table 1).

### 2.4. Drug-Resistance

*Pmr* is a dominant structural mutation in the coding region of small subunit of the rDNA, and it confers resistance to paromomycin (30 ng/*μ*L) [17]. *Chx* is a dominant mutation, which causes structural modification of large subunit of rDNA, and confers resistance to cyclohexamide (15 ng/*μ*L) [18, 19]. The dominant mutant allele *Mpr* is mapped to chromosome 2R, and confers resistance against 6-methylpurine(25 ng/*μ*L), which is a structural analog of adenine, and disrupts DNA synthesis in sensitive cells [20]. Since all drugs are lethal at the respective concentrations, the sensitive phenotype manifests as the presence of dead cells. A resistant phenotype is indicated by the presence of log-phase cells after 72 hours of exposure to a single drug [12] or 48 hours of exposure to two drugs applied simultaneously (this study). 

We verified the stability of drug resistance markers in the parental as well as the progeny strains listed in Table 1. Parental strains obtained from the stock center carry resistance alleles in their germline nucleus, but sensitive alleles in the somatic nucleus [15]. Because alleles in the somatic nucleus determine phenotypes, each parental strain is expected to show sensitivity to all drugs, including the one for which they carry resistance alleles in the germline. Progeny strains carry resistance alleles in their germline as well as somatic nucleus. Each progeny strain is expected to be resistant to only one drug, characteristic of the resistance allele in the germline nucleus of its parental strain. From a clonal culture of each strain (parental or progeny), we isolated 48 single cells and grew them asexually for 48 hours. Each of the 48 cultures was exposed separately to the three drugs, and scored for resistant phenotype. This allowed us to determine the frequency with which cells spontaneously acquired or lost resistance to one or more of the drugs. 

We also tested whether resistance markers affect the efficiency of each other. Efficiency of a resistance marker is calculated as the frequency of observing a resistant phenotype when expected. We performed all pairwise crosses between the 12 progeny strains (Table 1) to construct strains heterozygous for every pairwise combination of the markers. We picked 48 pairs from each cross, let their cultures grow asexually for 72 hours, and identified which cultures are sexually immature. Immaturity confirms that the pair mated successfully and exchanged the resistance markers (see below). We scored for dual drug resistance of the immature cultures. The extent of association between confirmed immaturity and the presence of dual drug resistance provides the efficiency of drug resistance markers in the presence of each other.

### 2.5. Mate Choice Assay

We used mate choice assays to quantify pairing frequency between the sexes II, IV, V, and VII. Every assay was performed between three sexes, each sex carrying a different drug-resistant genotype (Figure 1(b)). To create such a triplet of sexes, we used one progeny strain of each parent (Table 1, Figure 1). Thus, drug resistance allowed identification of morphologically indistinguishable sexes and quantification of the number of pairs formed between each of the three sexes.

Starvation is necessary to induce mating between sexes. At the beginning of every assay, we starved three sexes (i.e., the respective progeny strains) separately and adjusted the density of each to 2 × 10^5^ cells/mL. We mixed about equal numbers of the three sexes (6.6 × 10^3^ cells per sex) to a total density of 2 × 10^5^ cells in 1 mL (0.33 mL per sex). We define *T0* as the time at which we mix the sexes. Pairs start forming at ~3 hours (*T3*) after mixing. A pair takes between 10 and 12 hours to complete mating and then separates [21]. Before separation, the two sexes involved in a pair reciprocally exchange haploid genomes, including the drug-resistance markers. After separation, the partners (now called progeny) have dual drug-resistance, characteristic of the sexes involved in the pair. After 24 hours (*T24*), mostly single cells are observed, indicating separation of all pairs.

For every triplet, the mate choice assay was performed under a strict competition regime by providing limited time for choosing a mate. Thus, we picked 96 mating pairs early during mating process (*T6.5*). We put each pair in an individual drop of 1% PP medium, and grew them asexually for 48 hours. We replicated each drop-culture into 2% PP medium containing each drug separately and into the pairwise combinations of the three drugs. If the pair mated successfully, its culture will have dual drug resistance characteristic of the two sexes in the pair and is expected to grow in the respective drug combination. If mating was unsuccessful, its culture will grow in the presence of two of the individual drugs, but not in any pairwise combination of drugs. This allowed us to identify sexes involved in successful as well as unsuccessful pairing. We performed four replicates of every trial. For every triplet, we also repeated the mate choice assay giving the cells unlimited time to choose a mating partner by testing the dual-drug resistance of single cells isolated at *T24* (data not shown).

In addition to the drug resistance markers, we used immaturity tests to verify whether the isolated pairs successfully completed mating [16]. Mating partners in a pair separate after about 10 to 12 hours. If genetic exchange was successful, the separated partners are now progeny cells, which are sexually immature and show no pairing with any of the seven mating types until they reach sexual maturity (~100 asexual divisions). In an unsuccessful mating, the separated partners retain their sexual maturity. Hence, soon after separation, they are able to form pairs with a compatible mating type. We tested cultures of each pair for immaturity by mating tests with the parental strains within 5 days after picking mating pairs.

### 2.6. Differential Viability of Strains

Significant differences in viability of mating pairs between the progeny strains would skew our estimates of pairing frequencies. Thus, we conducted a separate experiment to test how frequently mating pairs between progeny strains died before scoring for dual resistance. We made every pairwise cross (total 36 crosses) between the 12 progeny strains (Table 1), and isolated 46 pairs at 6.5 hours after mixing the two progeny strains. We scored the survivorship as the proportion of viable pairs in 1% PP at 48 hours after-isolation. This procedure was replicated twice. We used these viability data to correct the estimates of the pairing frequencies in mate choice trials. We divided the number of pairs observed between two sexes in a mate choice trial by the viability estimate for the corresponding pair of progeny strains (Table  S1 available online at doi:10.1155/2012/201921). We then normalized the corrected numbers to 96: the total number of pairs isolated in each mate choice trial. This gave us an estimate of the actual number of pairs that likely formed between the two sexes. We used these normalized estimates of pairing frequencies in the analyses of mating biases. 

### 2.7. Statistical Analysis

In every trial conducted on a triplet of sexes, three types of pairs can be formed between the three sexes (Figure 1(b)). Under random mating, we expect 1/3 of total pairs to be of each type. We used goodness-of-fit tests to determine if mating was random within a trial. Also, we used mixed-effect ANOVA to analyze if mating was random in a triplet (i.e., pooling the data for all trials conducted for a given triplet). The experimental design (Figure 1(b)) contained one fixed factor (sex) and one random factor (drug resistant genotype). All statistical analyses were performed in *R*.

## 3. Results

Our aim was to investigate whether preferential mating occurs among sexes, when more than two sexes are present in a population. Hence, we quantified biases in pair formation between four (of seven) sexes of *T. thermophila*. We created experimental populations containing three sexes each and used drug resistance markers to identify how often the various sexes formed mating pairs.

### 3.1. Drug-Resistant Alleles Serve as Reliable Genetic Markers

We determined the reliability of the drug resistance alleles as genetic markers and found that they are stable against spontaneous mutations. The frequency of mutations conferring a loss or gain of resistance was below the limit of detection for all strains listed in Table 1 (data not shown), that is, we found no such spontaneous mutations. We also found the efficiency of the markers in conferring resistance to be 100% (data not shown) because only those strains that were immature showed dual drug resistance. Thus, the three resistance alleles are reliable genetic markers in the genetic backgrounds used in our experiments. While we did not explicitly test the neutrality of these markers towards fitness (growth rate) of the strains, at no step in our mate choice assays was there an opportunity for fertility selection. We did not, however, observe any obvious differences between growth rates of the progeny strains with different markers. 

### 3.2. Viability of Mating Pairs Is Contingent upon the Strains Forming the Pairs

We measured viability of mating pairs as the proportion of total pairs that survived and grew 48 hours after isolation in growth medium. We found that some mating pairs did die more frequently than others (one-way ANOVA, *P* < 2.2*e* − 16). This indicates that viability of the pairs is contingent upon the progeny strains forming the pairs (Table  S1). We could not associate the observed mortality with the presence of any particular resistance marker, but rather conclude that viability is a property of the specific genetic backgrounds of the strains. 

### 3.3. Mating Is Random with respect to Sex

Each experimental trial (Figure 1(b)) contained three sexes in equal proportion, which ensures equal mating opportunity for each sex. Thus, under random mating, the three types of pairs that could be formed among the three sexes should be represented at an equal frequency of ~0.33. We tested this null hypothesis for every trial using a goodness-of-fit *G*-test and found evidence of nonrandom mating (*P* < 0.05) in many trials but without a strong bias towards any particular sex (data not shown). The three sexes within a trial, however, had different genetic backgrounds. Hence, the apparent deviations from random mating may not represent mating preference with respect to sex of an individual but instead reflect an effect of genetic backgrounds on pairing frequencies in a trial. The genetic backgrounds used in our experiments represent a randomly chosen subset of the available drug resistant backgrounds; therefore, any effect of the genetic background on pairing frequencies may be specific to the strains we used, and must be isolated from the effect of the sexes. 

**Figure 2:**
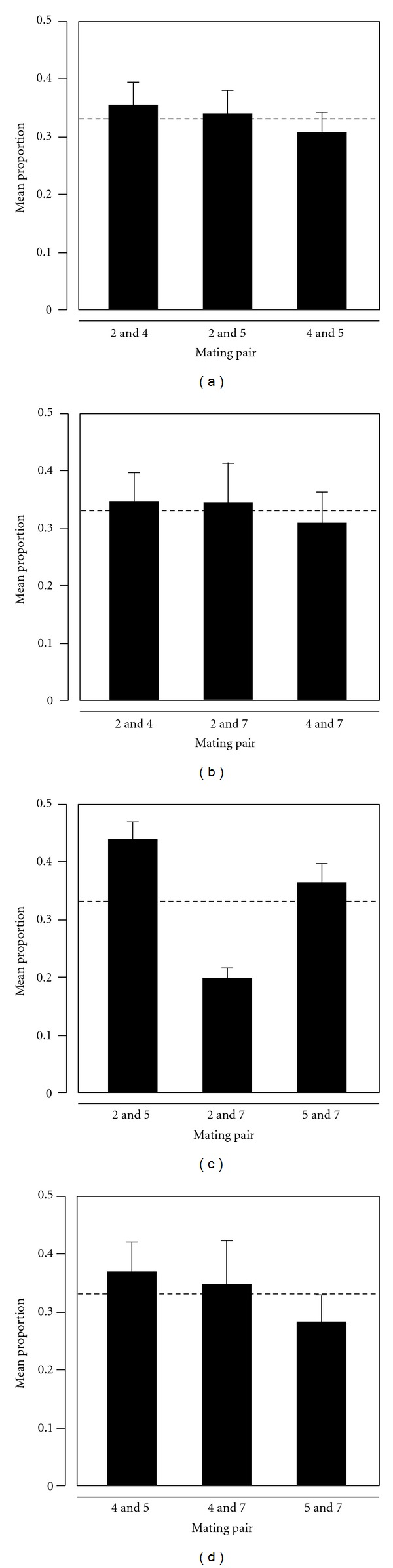
Random mating among sexes in *T. thermophila*. Bar charts show the relative frequency of pairs formed between sexes. Sexes are represented in Arabic numbers. Panels (a): triplet 2, 4, 5; (b): triplet 2, 4, 7; (c): triplet 2, 5, 7; (d): triplet 4, 5, 7. *y-axis* shows the mean proportion from 12 replicates for each pair type, averaged over the three trials for a given triplet (Figure 1(b)). A total of 1152 pairs (*N* = 96 pairs per trial X4 replicates per trial X3 trials per triplet) were analyzed for each triplet. Dashed line indicates the proportion of pairs (~0.33) expected under random mating. Error bars indicate standard error. All triplets show evidence of random mating with respect to sex (mixed-effect ANOVA *P* > 0.05, Table 2).

For every triplet of sexes, we had conducted three trials in which the drug resistant, genetic backgrounds alternated among the three sexes (Figure 1(b)). If sex of an individual was driving biases in pairing frequencies observed within a trial, we would expect a consistent bias towards a particular pair of sexes across the three trials irrespective of the associated genetic backgrounds. On the other hand, if the sexes exhibit no preferences, the pairing frequencies across the three trials will reflect random mating with respect to sex. To investigate any consistent effects of sex (with alternating genetic backgrounds) on pairing, we analyzed mating biases by pooling across the three trials conducted for a given triplet of sexes. A mixed effect ANOVA indicated no significant effect of the sex on the dynamics of pairing in each triplet of the sexes (Table 2, Figure 2). This suggests that mating was indeed random with respect to sex of an individual, and that the biases apparent in pairing frequencies in individual trials likely represent an effect of the genetic background rather than preferences among the sexes. Nonpreferential mating, that is, random mating with respect to sex, was verified in assays conducted at *T24* (data not shown).

## 4. Discussion

Nonrandom mating is pervasive in nature, occurring in various ways, and resulting in differing evolutionary consequences [1]. In contrast, our results suggest that *T. thermophila *mate randomly among each other with respect to sex. This lack of mate preference was apparent even under strict competition for mates imposed by limited time to find a mating partner, and subsequently when time restriction was removed.

Sexes of *T. thermophila* are self-incompatible, that is, cells of the same sex never form mating pairs, but each of the seven sexes can pair with all the other (i.e., six) sexes. Thus, even though all sexes look morphologically alike, they are somehow able to distinguish self from nonself. Our analysis suggests that beyond this basic distinction, most sexes of *T. thermophila* are unable to differentiate the various nonself sexes. Thus, the “choice” in this multisex system is binary, that is, it is about whether or not to form a mating pair rather than which of the available sexes to form the pair with. Our results are consistent with the previous finding that the sexes functionally substitute each other during costimulation—the stage immediately prior to mating pair formation [5]. Although it used a similar experimental design, the previous study did not allow quantification of mating preference of the four sexes. Our experimental design allowed us to verify that none of the sexes II, IV, V, and VII show bias for mating among each other when presented simultaneously with a choice between two compatible sexes. This could be possible if the ligands characterizing various sexes diverged to be sex-specific but still share a common receptor, enabling the sexes to replace each other functionally. Alternatively, random mating found in our study may reflect presence of a unique receptor for each sex-specific ligand and thus lack of competition between the sexes. 

Neither the molecular differences between the sexes nor the molecular interactions responsible for pairing are known in *T. thermophila*. Interaction between a sex-specific ligand on the surface of one sex and the receptor on the surface of the other sex is hypothesized to be the underlying mechanism of pairing between sexes, but awaits empirical support [6]. If pairing indeed results from such molecular interactions, then we suggest that affinities between the sex-specific ligands and receptor(s) may be of equal strength, leading to random mating between the sexes. It is generally observed in other unicellular species that the affinities between sex-specific molecules rarely determine the intraspecific mating propensities. For instance, the unique pheromones secreted by the two mating types in the yeast *Saccharomyces cerevisiae* bind to specific receptors displayed by the opposite mating type. Variation in the amount of pheromone produced, but not in the affinity to the receptor was found to be the basis of nonrandom mating [3]. In the ciliate *Euplotes raikovi*, sex-specific pheromones and receptors characterize the multiple sexes, which show variable mating interactions among each other beyond self-/non-self-distinction. Biased mating among the multiple sexes is a function of amount of pheromone produced such that higher secretion translates into higher mating success for any given sex. Thus, factors other than molecular affinities largely determine mating propensities among sexes in a population of this species [22]. Also, affinities between sex-specific molecules play a minimal role in interspecific mating interactions. For instance, although recognition of opposite mating type occurs and interspecific hybrids sometimes form between the two closely related yeasts *S. cerevisiae* and *S. paradoxus,* mating takes place preferentially with conspecifics and is more efficient and frequent [10, 23]. The overall genetic background rather than species-specific pheromone-receptor affinities are largely responsible for mating selectively within species. Thus, unlike in anisogamous plants and animals, prezygotic reproductive barriers may rarely occur at the pheromone/receptor level [11] in isogamous unicellular eukaryotes, allowing extensive diversification of mating types within species. 

The genetic background may also affect viability in *T. thermophila.* For example, pairing with the strain 5C always resulted in low viability (Table  S1) for reasons that are yet unclear and may involve genetic incompatibilities, which could be investigated in future studies. Although mating is random with respect to sex, the effect of genetic background on viability implies that some sexes may contribute to the gene pool more than the others, contingent upon the genetic background they are associated with. The effect of a sex and its genetic background on mating propensities could be explored in future studies using a full factorial design involving six (instead of three) trials for every triplet of sexes (Figure 1(b)).

Natural populations of *T. thermophila* are likely patchy, thus due to self-incompatibility and the inability to switch sexes, finding a compatible mate may be difficult ([24–26], P. Doerder, pers. comm.). Random mating with respect to sex creates the highest possible opportunity for mating, contingent upon the sex ratio, the relative frequencies of multiple sexes, in the population. High frequency of sex has been documented in natural populations of *T. thermophila *[25–27]. Thus, when searching for mates is costly, random mating with respect to sex is likely to be advantageous by avoiding further delays in initiating mating. It is unclear, however, why self-incompatibility would be maintained under such a scenario, though it is possible that high levels of inbreeding depression select for the maintenance of self-incompatibility. Transitions to self-compatibility in *Arabidopsis* have been linked to mate-limiting conditions [28]. Mating between cells of the same sex and other selfing strategies, including autogamy, are also observed in many species of ciliates and may reflect ways to avoid the cost of finding a mate [7, 29]. Alternatively, presence of multiple sexes may compensate, at least partially, for the cost of self-incompatibility. Very few data exist to test the correlation between the number of sexes and the capacity to transition to self-compatibility in ciliates.

The demonstration of random mating with respect to sex verifies a key assumption of our model on sex ratio evolution in *T. thermophila *[30]. Although violating the assumption of random mating would not change the equilibria predicted in this model, it would change the approach (time and the trajectory) of the populations to those equilibria. We studied mating preferences of the four sexes specified by a single sex determining allele (*mat-*2). The analysis presented here does not rule out the possibility of finding nonrandom mating between sexes specified by different *mat *alleles, which may lead to assortative mating with respect to the genotypes at the *mat *locus in *T. thermophila*.

Discovery of mating bias among sexes in *T. thermophila *could have delivered insights into affinities between sexes at the molecular level and facilitated predictions about the structural differences between the sex-specific molecules, which are currently unknown. Our results emphasize the need to decipher the molecular machinery that enables random mating among multiple sexes.

## Figures and Tables

**Table 1:** *T. thermophila* strains. All strains are whole-genome homozygotes and carry the *mat-2* allele at the sex determination locus [12].

Parental strain^a^	Progeny strain^b^	Mating type (sex)	Drug resistance marker in germline (in soma)^c^
CU438.1		IV	Pm-r (Pm-s)
	CU438.1-2	II	Pm-r (Pm-r)
	CU438.1-4	IV	Pm-r (Pm-r)
	CU438.1-5	V	Pm-r (Pm-r)
	CU438.1-7	VII	Pm-r (Pm-r)
CU428.2		VII	Mp-r (Mp-s)
	CU428.2-2	II	Mp-r (Mp-r)
	CU428.2-4	IV	Mp-r (Mp-r)
	CU428.2-5	V	Mp-r (Mp-r)
	CU428.2-7	VII	Mp-r (Mp-r)
CU427.4		VI	Cy-r (Cy-s)
	CU427.4-2	II	Cy-r (Cy-r)
	CU427.4-4	IV	Cy-r (Cy-r)
	CU427.4-5	V	Cy-r (Cy-r)
	CU427.4-7	VII	Cy-r (Cy-r)

^a^Strains obtained originally from the *Tetrahymena* stock center are derived from the inbred strain B upon mutagenesis (P. Bruns, pers. comm.). These strains were used to construct drug resistant progeny strains of various mating types.

^b^Progeny strains were generated using genomic exclusion [13]. All progeny strains show resistance to the respective drug owing to the resistance alleles they inherited from the germline of their parental strain.

^c^Drugs are abbreviated: Pm: paromomycin, Mp: 6-methylpurine, Cy: cyclohexamide. Resistant phenotypes are indicated by “-r” and sensitive phenotypes by “-s”.

**Table 2:** Pairing frequencies within triplets of sexes were analyzed by ANOVA. The *F* ratios and *P* values refer to the effect of sex.

Triplet^a^	*F* ratio	*P* value
**2** **, 4, 5**	0.7209	0.5403
**2, 4, 7**	0.3372	0.7323
**2, 5, 7**	4.8167	0.0861
**4, 5, 7**	0.3385	0.7315

^a^Sexes are indicated as Arabic numbers instead of Roman numerals for convenience.
